# Preparation and Characterization of a Novel Artemisia Oil Packaging Film and Its Application in Mango Preservation

**DOI:** 10.3390/foods12152969

**Published:** 2023-08-07

**Authors:** Xiaohan Meng, Ze Lv, Tianzhen Jiang, Yifei Tan, Shaoyang Sun, Jianguo Feng

**Affiliations:** College of Plant Protection, Yangzhou University, Yangzhou 225009, China

**Keywords:** soybean protein isolate, gelatin, Artemisia oil, mango preservation, active packaging film

## Abstract

In this work, a new food packaging film was synthesized via blending Artemisia oil (AO) into soybean protein isolate (SPI) and gelatin (Gel) for the postharvest storage of mango. The morphological architecture and mechanical properties of the films were characterized using scanning electron microscopy (SEM), atomic force microscopy (AFM), Fourier transform infrared spectroscopy (FTIR), X-ray diffractometer (XRD), and other technologies. The results show that the prepared films had relatively flat surfaces with good mechanical properties. AO enhanced the light-blocking ability of the film, increased the hydrophobicity, and affected the moisture content and water solubility of the film to a certain extent. Furthermore, the antioxidant performance and antifungal (*Colletotrichum gloeosporioides*) capacity of the films increased with higher AO concentration due to the presence of the active components contained in AO. During mango storage applications, the films showed good freshness retention properties. The above results indicate that SPI–Gel films containing AO have excellent physicochemical and application properties and have great potential in the field of food packaging.

## 1. Introduction

Mango (*Mangifera indica* L.) is of the genus Mango in the family Anacardiaceae. It has an attractive appearance, rich aroma, delicious taste, and high nutritional value, so it enjoys the honor of being the “king of tropical fruits” [1,2]. However, as a tropical fruit, mango is metabolically active after harvest, resulting in weight loss, the conversion of starch to sugar, texture softening, and decay [3]. In addition, some plant diseases (common ones such as anthracnose) can cause outbreaks of negative effects during mango storage, rendering the fruit inedible [4]. These factors significantly reduce the economic and nutritional value of mangoes. Thus, effective postharvest preservation of mango has always been an essential economic and health issue.

Currently, in the field of fruit preservation, active food packaging relying on biocompatible films is a prospective approach due to their ability to reduce the respiratory intensity, transpiration, and oxidation rates, and such films have been increasingly used to maintain the quality of fruits during storage [5]. In the case of active food packaging production, the harmful effects of synthetic chemicals on consumer health and the environment are well known, and the use of natural active compounds is always preferred [6]. Hence, the development of biodegradable, environmentally friendly, green, and safe packaging materials as alternatives to traditional synthetic food packaging is gaining increasing attention.

Typically, biopolymers such as soybean protein isolate, cellulose, starch, sodium alginate, pectin, etc., are often blended with nanoparticles, essential oils, polyphenols, etc. to prepare composite films via the casting method [7]. Soybean protein isolate (SPI) is a cheap and safe renewable resource with diverse globulin fractions widely used in bioplastics and films for food applications. Due to its network structure formed by the interactions of intramolecular and intermolecular hydrophobic and disulfide bonds, SPI exhibits excellent mechanical properties but poor barrier performance and tends to absorb a high amount of moisture after fabricating the films [8]. Gelatin (Gel) is a natural colloidal macromolecular material derived from animal skin, bone, and connective tissue, which is abundant and easy to form films. In view of Gel’s excellent hydrophilicity and barrier properties to oxygen, carbon dioxide, and light, it has been widely applied as a packaging material to protect food from drying and prevent its exposure to external light and gases [9]. Intermolecular hydrogen bonds can be formed between SPI molecules and carbonyl, hydroxyl, and amino groups in Gel molecules, which ensures that crosslinking them can form a composite film with excellent mechanical and barrier properties [10].

For the purpose of equipping packaging films with some biological activity, active food packaging films are usually prepared by incorporating some active ingredients into the film-forming solution in various ways [11]. Essential oils (EO) are regarded as the best choice of active ingredient because of their high antimicrobial activity and safety. Research has demonstrated that films containing EOs can effectively inhibit bacteria, stop spoilage, and extend the shelf life of food [12]. Artemisia oil (AO) is an active ingredient extracted from *Artemisia annua* L., a plant of the Asteraceae family, with a variety of biological functions such as antibacterial, antiviral, antifungal, antioxidant, and insecticidal activities which have been extensively applied in the fields of medical treatment, food preservation, and plant protection [13]. By uniformly distributing AO in the film-forming solution through high shear, biologically active packaging films can be obtained.

In this research, a crosslinked hybrid film based on SPI and Gel was fabricated via the casting method, and AO was incorporated into the film for fruit preservation purposes. While studying the structural properties and physicochemical properties of the films, the antifungal and antioxidant properties were explored and their effects on the postharvest storage quality of mango were observed at room temperature. This work contributes to the evolution of novel active food packaging and provides new strategies to extend the preservation of fresh fruits and agricultural products.

## 2. Experimental Section

### 2.1. Materials and Chemicals

Soybean protein isolate (SPI, 99%), gelatin (Gel, 100 Bloom), glycerol, and sodium hydroxide (NaOH) were all acquired from Aladdin Biochemical Technology Co., Ltd. (Shanghai, China). Anhydrous calcium chloride (CaCl_2_) was bought from Lianyungang Runtai Chemical Co., Ltd. (Lianyungang, China). All chemicals were of analytical grade and utilized as received. Artemisia oil (artemisinin content 60%) was purchased from Jiangxi Xinsen Natural Plant Oil Co., Ltd. (Ji’an, China).

The mangoes (Tainung No.1, late green ripe stage) used in the experiment were procured from the local fruit market. *Colletotrichum gloeosporioides (C. gloeosporioides)*, the causal pathogen of anthracnose of mango, an important disease on mango which causes black spots on mango, resulting in rot and economic loss [14], was provided by the Plant Pathology Laboratory, College of Plant Protection, Yangzhou University.

### 2.2. Synthesis of Films

Control films were formulated through the following method: 5 g SPI and 2 g glycerol were added to 100 mL deionized water, stirred continuously using a magnetic mixer until the solid was completely dissolved, and the pH was adjusted to 9–10 via dropping 1.0 mol L^−1^ NaOH solution and then stirred at 70 °C for 30 min. In addition, 5 g of Gel was dissolved into 100 mL 55 °C deionized water to obtain a 5% Gel solution. Finally, the above two solutions were mixed in equal amounts. To avoid variations in thickness, the solution was evenly dispersed in plastic Petri dishes and put in a vacuum-drying oven at 55 °C overnight. The final control film obtained was recorded as S-G.

The films containing AO were prepared in essentially the same way as described above. In short, different amounts of AO (1%, 2%, 3%, *w/v*, depending on the total film forming solution) were mixed into the film-forming solution of blank film and then homogenized at 10,000 rpm for 5 min using an FA25-Digita high shear dispersing emulsifier (Fluke, Shanghai, China) and subjected to ultrasonication for 30 min to eliminate air bubbles. The films obtained by laying down and drying were labeled as S-G-1, S-G-2, and S-G-3, respectively.

### 2.3. Structural Characterization

#### 2.3.1. Scanning Electron Microscopy (SEM)

The film was visualized via SEM (S-4800, Tokyo Hitachi Co., Ltd., Tokyo, Japan) for surface morphology and cross-section morphology. Before analysis, the films were frozen in liquid nitrogen and cracked to obtain the cross-section to be observed.

#### 2.3.2. Atomic Force Microscopy (AFM)

The surface appearance of the films was observed via AFM (Shimadzu, Tokyo, Japan) to reflect the roughness of the individual films.

#### 2.3.3. Fourier Transform Infrared Spectrometer (FTIR)

The FTIR spectra of prepared films and Artemisia oil were obtained using a Fourier transform infrared spectrometer (Agilent Cary 630, Agilent Technologies, Inc., Santa Clara, CA, USA) scanning in the range from 4000 to 400 cm^−1^ with a nominal resolution of 4 cm^−1^.

#### 2.3.4. X-ray Diffraction (XRD)

A polycrystal X-ray diffractometer (D8 Advance, Bruker Ltd., Karlsruhe, Germany) was applied to examine the crystal structure of the films at angles ranging from 5° to 85°.

### 2.4. Physical Properties

#### 2.4.1. Thickness

The thickness was detected using an EVERTE digital micrometer (Shanghai Aladdin Bio-Chem Technology Co., Ltd., Shanghai, China). Ten positions were measured randomly for each film, and the final average was used to calculate other mechanical and physical properties. Moreover, an LSM 700 3D Laser Scanning Microscope (Zeiss, Jena, Germany) was also used to measure the thickness of each film.

#### 2.4.2. Mechanical Properties

After placing the film in 50% relative humidity at 25 °C for 48 h, the film was cut into 1 cm × 6 cm rectangular samples for measurement. The film strips were tested on an STX200 Universal Testing Machine (Yishite, Xiamen, China) to obtain the tensile strength (TS), elongation at break (EAB), and Young’s modulus (YM) of the film samples, with a crosshead speed of 5.00 mm min^−1^. The TS, EAB, and YM were calculated using the following equation [15]:

(1) $$\mathrm{TS}=\frac{F}{x\times W}$$

(2) $$\mathrm{EAB}=\frac{\Delta L}{L_{0}}\times 100\%$$

(3) $$\mathrm{YM}=\frac{F\times L_{0}}{A\times \Delta L}$$

where F is the film fracture stress (N), x denotes the thickness of the film (mm), W denotes the width of the film (mm), ∆L denotes the length added when the film fractures (mm), and L_0_ denotes the initial film length (mm). Three replicates of each test were conducted for each film.

#### 2.4.3. Color

The L* (lightness), a* (red/green), b* (blue/yellow), and Δ*E* (total color difference) values of film samples were measured with a colorimeter (CS-420, Hangzhou, China), with the background color of CIELAB-grade measurement color. Measurements were taken five times per film. The Δ*E* was calculated according to the following equation:

(4) $$\Delta E=\sqrt{{\left(L^{*}-L\right)}^{2}+{\left(a^{*}-a\right)}^{2}+{\left(b^{*}-b\right)}^{2}}$$

#### 2.4.4. Light Transmittance

The film was measured via a UV–Vis–NIR absorption spectrometer (Cary 5000 Varian, Tampa, FL, USA) to determine its light-blocking capacity. The size of the films was cut to 5 cm × 5 cm, and the spectral acquisition range was 200 to 800 nm. The *opacity* at 600 nm was calculated according to the equation [16]:

(5) $$Opacity=\frac{-logT_{600}}{x}$$

where *T*_600_ is the percentage transmittance at 600 nm, and *x* is the film thickness (mm).

#### 2.4.5. Water Vapor Permeability (WVP)

The water vapor barrier properties of the film samples were calculated using the gravimetrical method [17]. Briefly, 5 g CaCl_2_ was put into a conical flask, which was sealed with the prepared film. The conical flask was stored at 100% relative humidity and 25 °C, and the conical flask’s mass was weighed daily for 3 days. The *WVP* was calculated using the equation:

(6) $$WVP=\frac{W\times x}{t\times A\times \Delta P}$$

where *W* denotes the weight increase in the conical flask (g), *x* denotes the thickness of the film (m), *t* denotes the time taken for weight to increase (s), *A* denotes the permeable area of the film (m^2^), and ∆*P* denotes the partial vapor pressure. Three replicates of each test were conducted for each film.

#### 2.4.6. Water Contact Angle Measurements

The water contact angle can reflect the hydrophobicity of the film surface, and the larger the contact angle, the stronger the hydrophobicity. The change in contact angle of water droplets on the surface of the films (1 cm × 4 cm) was recorded every second for ten seconds with a dynamic contact angle goniometer (Dataphysics, Germany) equipped with a high-speed CCD camera. The surface of each film was tested five times.

#### 2.4.7. Moisture Content (MC) and Water Solubility (WS)

In order to determine the moisture content and water solubility of the prepared films, the films were cut into 3 cm × 3 cm square samples, and the weight was recorded as *W*_0_ (g). Then, the samples were dried at 105 °C for 24 h, and when the weight was constant, the weighed weight was recorded as *W*_1_ (g). The moisture content (*MC*) of the films was calculated using the following equation [18]:

(7) $$MC\;\left(\%\right)=\frac{W_{0}-W_{1}}{W_{0}}\times 100\%$$

Water solubility (*WS*) is defined as the weight percentage of the soluble component of the film. After drying the prepared films, they were thrown into deionized water at 25 °C for 24 h and then taken out, followed by drying at 105 °C overnight to reach a constant weight, which was weighed and recorded as *W*_2_ (g). The *WS* of the sample was determined using the following equation [18]:

(8) $$WS\;\left(\%\right)=\frac{W_{1}-W_{2}}{W_{1}}\times 100\%$$

All measurements were performed in three replicate trials.

### 2.5. Application

#### 2.5.1. Antioxidant Activity

##### DPPH

The prepared films’ antioxidant activities were estimated via the scavenging rate of DPPH radicals [19]. Briefly, 40 mg cut film was placed in 6 mL ethanol to obtain the extract; then, 3 mL of the extract was taken and mixed thoroughly with 1 mL of 0.1 mM ethanol DPPH solution. The mixture was incubated in the dark for half an hour. The absorbance of the supernatant liquor at 517 nm was measured using a UV–Vis spectrophotometer (INESA, China). The *DPPH radical scavenging rate* is determined via the following equation:

(9) DPPH radical scavenging rate (%)=(1−ASAC)×100%

where *A_S_* denotes the absorbance of the *DPPH* radical solution mixed with the sample and *A_C_* denotes the absorbance of the initial *DPPH* radical solution. Three tests were performed on each specimen and the mean value was calculated.

##### ABTS

The ABTS was formulated as a 7 mM concentration aqueous solution, mixed with 2.5 mM potassium persulfate solution in equal amounts, and allowed to stand for 16 h. Then, the solution was diluted with ethanol until an absorbance of 0.70 ± 0.02 at 730 nm was reached, and the absorbance was recorded as *A*_0_. Moreover, 10 μL of film-forming solution was added to 990 μL of the diluted solution. After standing in the dark for 6 min, the absorbance was measured at 730 nm and recorded as *A*_1_. The standard curve was obtained with different concentrations of gallic acid solutions. Three tests were performed on each specimen, and the mean value was calculated. The ABTS radical scavenging activity was calculated via the following equation [20]:

(10) $$\mathrm{ABTS}\;\mathrm{radical}\;\mathrm{scavenging}\;\mathrm{activity}\;\left(\%\right)=\frac{A_{0}-A_{1}}{A_{0}}\times 100\%$$

#### 2.5.2. In Vitro Antifungal Activity

*Colletotrichum gloeosporioides (C. gloeosporioides)* was cultured on potato agar plates (90 mm) at 25 °C in the dark. After a certain period of time, the culture was transferred to a 4 °C environment as a fungal source for in vitro activity assays of the films. Then, the prepared groups of films (UV sterilized for 20 min, 5 mm in diameter) were placed symmetrically on potato agar plates with 5 mm mycelium plates and incubated in a dark incubator at 28 °C to evaluate the in vitro antifungal activity. Sterilized blank filter paper sheets (5 mm in diameter) were used as a control, and three replicates were set up for each group.

#### 2.5.3. Preservation and Storage of Mangoes

Mangoes of the same size and ripeness were randomly divided into five groups. Before formal experiments, all mangoes were soaked in 2.5% hydrogen peroxide water for 3 min, rinsed, and dried. Then, the mangoes were wrapped using S-G, S-G-1, S-G-2, and S-G-3 films, respectively, and those without film wrapping were set as blank control. Samples were placed in an incubator at 25 ± 1 °C and relative humidity of 55% ± 3%. The indicators of these mangoes were observed and recorded daily. Five replicates were set up for each group.

The mango yellow area was identified by the photo of the mango sample using Photoshop software and calculated as the percentage of yellow area in the whole mango peel area. When the mangoes all turned yellow, observation stopped.

*Weight loss* is an extremely important indicator of the change in the quality of mangoes during storage, which was calculated using the equation [21]:

(11) Weight loss (%)=(1−mm0)×100%

where *m* is the weight of the mango after a scheduled time (1 day to 7 days, every day), and *m*_0_ is the initial weight.

### 2.6. Statistical Analysis

Data from the experiments were expressed as mean ± standard deviation (SD). Statistical analysis of the data was conducted using the analysis of variance (ANOVA) software SPSS (IBM version 21).

## 3. Results and Discussion

### 3.1. Structural Characterization

#### 3.1.1. SEM

**Figure 1:**
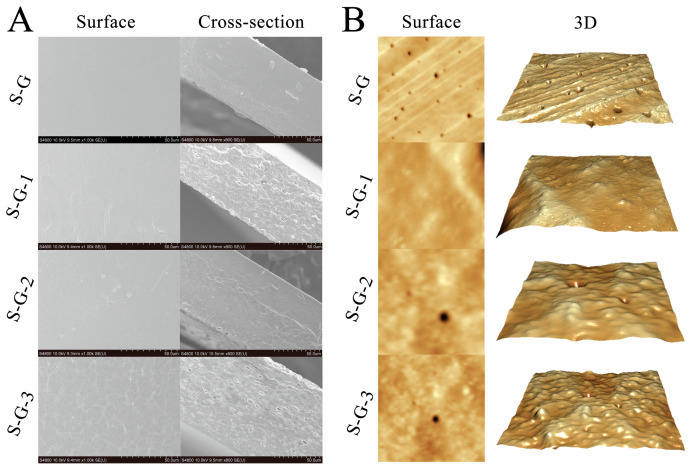
Surface and cross-section SEM images (**A**) and AFM images (**B**) of prepared films.

SEM images of the surface and fracture cross-sections of the films with different AO concentrations are shown in Figure 1A. The results show that the surface of the S-G film is continuous and flat, with no obvious bumps or pits, which might be connected to the appearance of ordered phases and a homogeneous protein crosslinked network structure. However, with the increase in the AO concentration, the film surface becomes relatively rough with respect to the S-G film, which might result from the agglomeration and flocculation of the AO dispersed within the film-forming solution during the drying process [22].

The cross-sectional part of the films revealed a rough structure composed of protein chains, which is also depicted in Figure 1A. The cross-section of the S-G film is relatively smooth and flat, indicating that the SPI and Gel formed a better fusion and crosslinking, which might be attributed to the formation of hydrogen bonds between them [23]. The cross-sectional roughness of the film increases gradually with the increasing AO concentration, and there was an increase in the number of particles, strips, and lipid droplet holes in the cross-section. This may be due to the increase in roughness influenced by hydrogen bonding and other interactions between the AO and SPI–Gel protein chains [24], which led to agglomeration in the three-dimensional network, in addition to the potential disruption of protein interactions in the film network due to the embedding of AO droplets [25].

#### 3.1.2. AFM

As shown in Figure 1B, AFM successfully mapped the typical surface morphology of the films with different AO concentrations. It is evident that the surface roughness of S-G and S-G-1 are comparable, indicating that adding a small amount of AO has no apparent effect on the surface morphology of the film. Nevertheless, with the increase in AO concentration, some bumps and depressions can be clearly observed on the surface of the films, and the higher the AO concentration, the more uneven the surface of the films. In addition, AFM observations are shown in Table 1, describing the roughness parameters R_a_ (the absolute value of the mean surface height’s deviation), R_z_ (maximum value in the z-direction), and R_q_ (the root mean square of the mean data plane height’s deviation) for different films. Through the comparison of the specific data in Table 1, R_a_, R_z_, and R_q_ tended to increase after the addition of AO, and S-G-1 is similar to S-G in all parameters, while S-G-2 and S-G-3 both show a significant increase in all parameters (*p* < 0.05), indicating that the increase in essential oil may result in an increase in film roughness [26,27], which is consistent with the results observed in Figure 1B.

#### 3.1.3. FTIR

**Figure 2:**
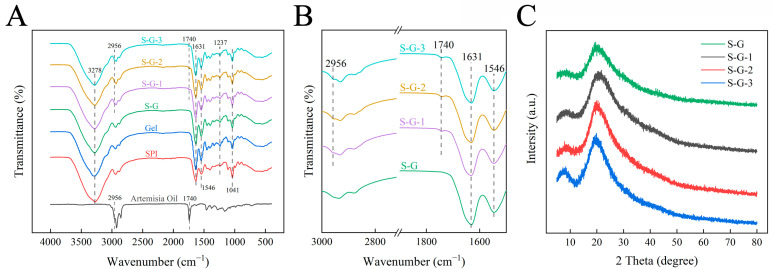
FT-IR (**A**), partial magnification (1500–3000 cm^−1^) FTIR (**B**), and XRD (**C**) of prepared films.

The FTIR spectra of the prepared films and AO are shown in Figure 2A. The impact of AO on the films is minimal, primarily because of the relatively low concentration of AO incorporated into the films compared with the high concentrations of SPI and Gel. As a result, the spectra of these prepared films exhibit similarity, a phenomenon similar to previous studies [28]. The broad characteristic band in the extent of 3000–3700 cm^−1^ refers to the stretching vibration of -OH. The main absorption peaks were associated with amide I (C=O stretching, 1631 cm^−1^), amide II (N-H bending, 1546 cm^−1^), and amide III (C-N stretching, 1237 cm^−1^) [29], which were used to track the crosslinking of film proteins. In addition, as shown in Figure 2B, the characteristic peaks of AO appeared in the spectra of S-G-1, S-G-2, and S-G-3, corresponding to the stretching vibration of C=O at 1740 cm^−1^ and C-H at 2965 cm^−1^, respectively [30], and the intensity of the peaks appeared to be slightly enhanced with the increase in the concentration of AO, suggesting that AO has relative compatibility with the film.

#### 3.1.4. XRD

The XRD spectra of each film in the range of 2θ = 5°–80° are shown in Figure 2C. The XRD spectra of the S-G film have relatively distinct derivative peaks around 2θ = 8.1° and 19.5°, which refer to the α-helix and β-sheet structures of the protein chain secondary structure, respectively [31]. After AO adding, no new peaks are appearing in the spectrograms of the films, which indicates that AO is compatible with the components of the films [32]. This is also due to the strong peaks of SPI and Gel and the low concentration of AO that limits the appearance of new peaks. It is noteworthy that the intensities of the derivative peaks gradually increase with increasing AO concentration, which is significantly higher than that of the S-G film, indicating an increase in crystallinity [33]. This may be due to the addition of AO involved in the interaction between SPI and Gel [34].

### 3.2. Physical Properties

#### 3.2.1. Thickness

**Figure 3:**
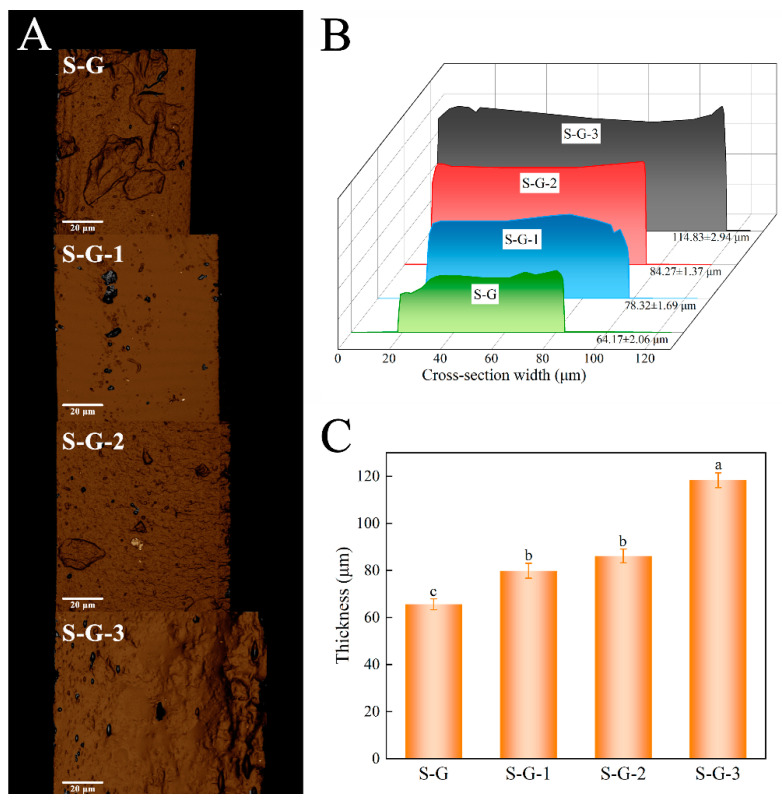
Three-dimensional cross-section structural conformation (**A**), cross-section width (**B**), and thickness measured by digital micrometer (**C**) of prepared films. Different letters for the same test parameter indicate significant differences among different groups (*p* < 0.05).

The results from the 3D laser scanning microscope and digital micrometer measurements are depicted in Figure 3. Figure 3A displays the 3D structural conformation of various film cross-sections, while Figure 3B illustrates the cross-section width observed using the 3D laser scanning microscope, directly indicating the thickness variations among the different films. The results indicate that the thicknesses of the S-G, S-G-1, S-G-2, and S-G-3 films are 64.17 ± 2.06 μm, 78.32 ± 1.69 μm, 84.27 ± 1.37 μm, and 114.83 ± 2.94 μm, respectively. These measurements are not significantly different from the values obtained using the micrometer (Figure 3C), and Figure 3C shows that low concentrations of AO have a significant effect on film thickness as early as at the *p* < 0.05 level, and there is also a significant difference in thickness when AO reaches a high concentration (3%) relative to low concentrations.

It can be seen that the thickness of the film gradually rises as the concentration of AO increases. This can be attributed to the physical bonding and association between the film and AO, as well as the formation of hydrophobic AO droplets in the process of homogenization of the film-forming solution, which may also contribute to the increase in film thickness [35]. Consequently, the results suggest that an increase in AO concentration causes a corresponding increase in film thickness.

#### 3.2.2. Mechanical Properties

**Figure 4:**
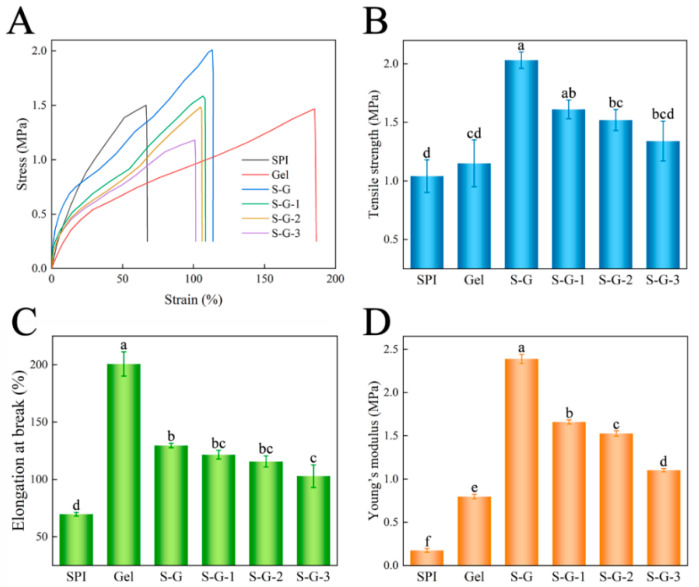
Stress–strain curve (**A**), tensile strength (**B**), elongation at break (**C**), and Young’s modulus (**D**) of the prepared films. Different letters for the same test parameter indicate significant differences among different groups (*p* < 0.05).

The assessment of mechanical properties is a necessary indicator of quality when it comes to food packaging films. Tensile tests yielded typical stress–strain curves, as shown in Figure 4A, and the variations in the tensile properties of the films are shown in Figure 4B,C. The results show that Gel films and their composite films have excellent deformation elongation, except for SPI films alone. The tensile strength of the S-G film increased significantly (about twice as much as that of the SPI and Gel films) when the SPI and Gel were mixed and crosslinked, and the tensile strength decreased with the increase in AO concentration, but there was no significant effect (*p* < 0.05). However, the increase in strength was at the expense of elongation, and the elongation at the break of the S-G film decreased significantly compared with the Gel films, and the elongation at the break of the S-G film was also slightly decreased by the addition of AO [36], but there was no significant effect (*p* < 0.05). Furthermore, the results concerning Young’s modulus (Figure 4D) indicate that the S-G composite film exhibits higher deformation resistance and rigidity compared with the individual SPI and Gel films [37]. Upon addition of AO, the rigidity of the film decreases with the increasing AO concentration.

#### 3.2.3. Color

**Figure 5:**
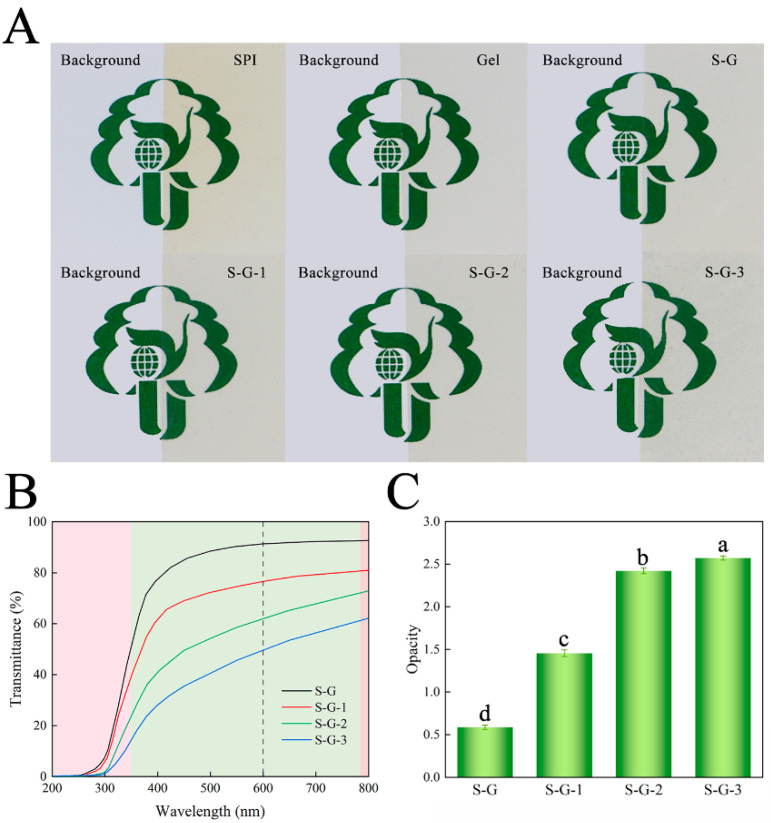
Appearance diagrams (**A**), light transmission (**B**), and opacity value (**C**) of prepared films. Different letters for the same test parameter indicate significant differences among different groups (*p* < 0.05).

Surface color is a vital optical property that can influence consumers’ choice of packaged foods. The color parameters of the prepared films are shown in Table 2. Generally, within the range of 0–100, *L* values indicate a color transition from black to white, while *a* values between 80 and 100 represent a color shift from green to red. Similarly, *b* values between 80 and 70 suggest a color transition from blue to yellow. As the concentration of AO increases, the colors of the films change, the value of parameter L* decreases, and the parameters a*, b*, and ΔE increase, which suggests a gradual darkening of the films’ colors, but there is no significant effect (*p* < 0.05) [38]. This may be due to the low transparency of AO itself, which darkens the color of the films [39]. The above results are consistent with those presented in Figure 5A, where Gel diminishes the yellow color of the SPI, and the addition of AO gives the film color a darkening trend.

#### 3.2.4. Light Transmittance

Light transmittance affects the clarity of the film and, therefore, the sensory effect. Figure 5B,C shows the ultraviolet–visible (UV–Vis) transmittance and opacity of each film, respectively. Within the UV wavelength range of 200~350 nm, all the films have good UV-blocking performance, and the UV-blocking ability of the films is gradually enhanced with the addition of AO. In the visible wavelength band (350~780 nm), the light-blocking performance of the AO-added films were obviously superior to those of the S-G films, and the opacity at 600 nm was also significantly higher (*p* < 0.05), which is consistent with Figure 5A. The droplets formed by AO scatter the light, thus interfering with light transmission [40]. Packaging films that effectively block light could delay the oxidization and deterioration of food.

#### 3.2.5. WVP

The moisture content of fruits is an important criterion for consumers to consider; so, in this field of fruit preservation, the water vapor permeability is a key index for examining the quality of food packaging films. The WVP of the prepared films is presented in Table 2. Water vapor transport usually occurs in the hydrophilic part of the film-crosslinked network. Hence, the WVP is also influenced by the ratio of hydrophilic to hydrophobic components present in the film. Previous studies have shown that the incorporation of essential oils increases the WVP of the film [41]. Interfacial interactions between AO and film components resulted in a decrease in protein–water interactions, possibly allowing for the free passage of water molecules and thus increasing the WVP significantly (*p* < 0.05) [42]. Furthermore, the incorporation of AO can have a plasticizing effect on the film, which further weakens the interactions between proteins within the film structure [43], thus increasing the WVP.

#### 3.2.6. Water Contact Angle Measurements

**Figure 6:**
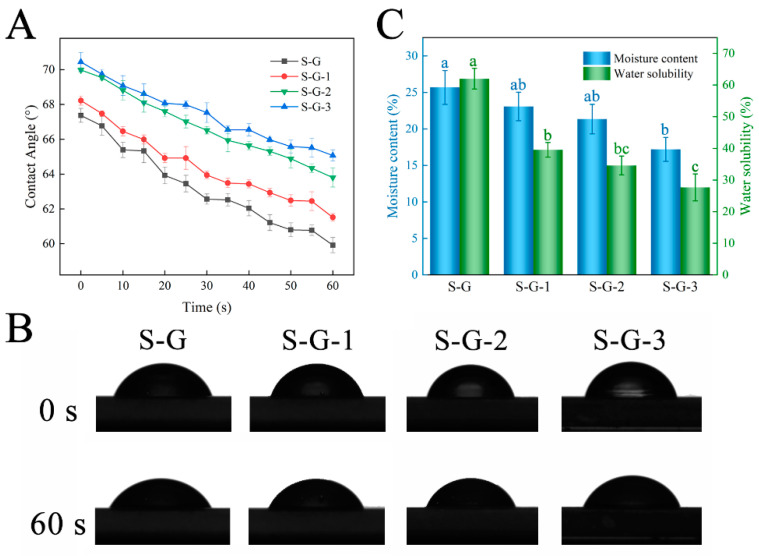
Water contact angle (**A**), transient photos (**B**), and moisture content and water solubility (**C**) of prepared films. Different letters for the same test parameter indicate significant differences among different groups (*p* < 0.05).

The water contact angle reflects the level of hydrophobicity of the material: the larger the contact angle, the more hydrophobic the material, and the other way around, the smaller it is. Figure 6A demonstrates the variation in contact angle with time, and Figure 6B presents the photographs of the water contact angle of different films at 0 s and 60 s. The results show that when SPI was crosslinked with Gel, the S-G film exhibited a relatively high contact angle, which was 67.37° at 0 s, which may be attributed to the existence of some hydrophilic groups, such as -OH and -COOH, in the Gel and SPI molecule [44]. Upon introducing AO, the water contact angle appeared to increase, reaching 68.22° for S-G-1, 69.98° for S-G-2, and 70.44° for S-G-3. This can be accounted for by the existence of numerous hydrophobic groups AO contains, which counteract the effect of hydrophilic groups in SPI and gelatin [45]. Consequently, the incorporation of AO enhanced the hydrophobic properties of the films.

#### 3.2.7. MC and WS

Moisture content and water solubility are essential indicators for evaluating food packaging films. As shown in Figure 6C, the MC of the S-G film was 25.68%, and with the progressive addition of AO, the MC exhibited a gradual decline, ranging from 23.08% to 17.19%. This observation can potentially be explained by two factors: firstly, the inclusion of AO may result in a decreased proportion of hydrophilic groups within the film; secondly, AO might disrupt the hydrated structure that forms between SPI and Gel [46]. The high WS of the S-G films (up to 62.02%) is due to the nature of SPI and Gel themselves, which both crosslink to form a hydrophilic polymer network through their interactions and thus exhibit favorable water solubility in aquatic environments [47]. However, the WS of the films decreased remarkably with increasing AO concentrations to 39.56%, 34.62%, and 27.67%, respectively (*p* < 0.05). The hydrophobic groups in the AO enhanced the water resistance of the films and increased the density of the molecular network in the films, and its hydrogen bonding interaction with the SPI-Gel protein molecules contributed to the formation of a strong structure that prevented the solubilization of the film components and reduced the water solubility [48].

### 3.3. Application

#### 3.3.1. Antioxidant Activity

##### DPPH

**Figure 7:**
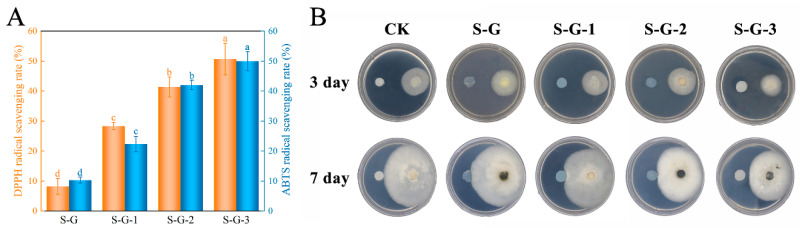
DPPH and ABTS radical scavenging rate (**A**) and in vitro antifungal test (**B**) of prepared films. Different letters for the same test parameter indicate significant differences among different groups (*p* < 0.05).

As seen in Figure 7A, the higher DPPH radical scavenging rate with increasing AO concentration was mainly attributed to the abundant ketones, alkenes, and sesquiterpenoid components with antioxidant properties contained in AO [49]. The DPPH radical scavenging rate of S-G films was only 8.23%, which increased to 23.08% and, finally, 50.66% after the addition of AO, indicating that AO has good antioxidant potential and significantly enhanced the films’ antioxidant properties (*p* < 0.05).

##### ABTS

The result of the ABTS radical scavenging capacity was comparable to the DPPH. With the increase in the AO concentration, ABTS radical scavenging also significantly increased (*p* < 0.05). The presence of AO raised the ABTS of S-G films from 10.32% to 49.97%, which was mainly attributed to the presence of antioxidant substances such as terpenoids and ketones in AO, which act as free radical receptors and terminate the oxidation reaction [50]. The above results indicate that the incorporation of AO ensured that the films met the requirements for food preservation.

#### 3.3.2. In Vitro Antifungal Activity

The antifungal activity of S-G, S-G-1, S-G-2, and S-G-3 films against *C. gloeosporioides* was determined, and the results are shown in Figure 7B. The inhibitory activity was observed on day 3 and day 7 of the experiment. On the third day, the control mycelium grew normally, and the S-G, S-G-1, and S-G-3 film-treated mycelia temporarily showed normal growth patterns, whereas the growth of the S-G-3 film-treated mycelium had been inhibited. By day 7, compared with the control group, the mycelium of S-G film treatment grew even more vigorously and had already grown onto the film, which may be due to the fact that SPI and Gel provided an additional source of C and N for the growth of mycelium [51], and the growth of the mycelia of both the S-G-1 and S-G-2 film treatments was inhibited when they were about to come into contact with the films, while the mycelium of the S-G-3 film treatment was inhibited the most obviously. This may be due to the fact that some components with antifungal activity volatilized after AO reached a certain high concentration, which effectively inhibited the growth of the mycelium [52]. The above result demonstrated that the incorporation of AO could make the films capable of preventing and treating *C. gloeosporioides*, and the higher the AO concentration, the more powerful the antifungal activity.

#### 3.3.3. Preservation and Storage of Mangoes

**Figure 8:**
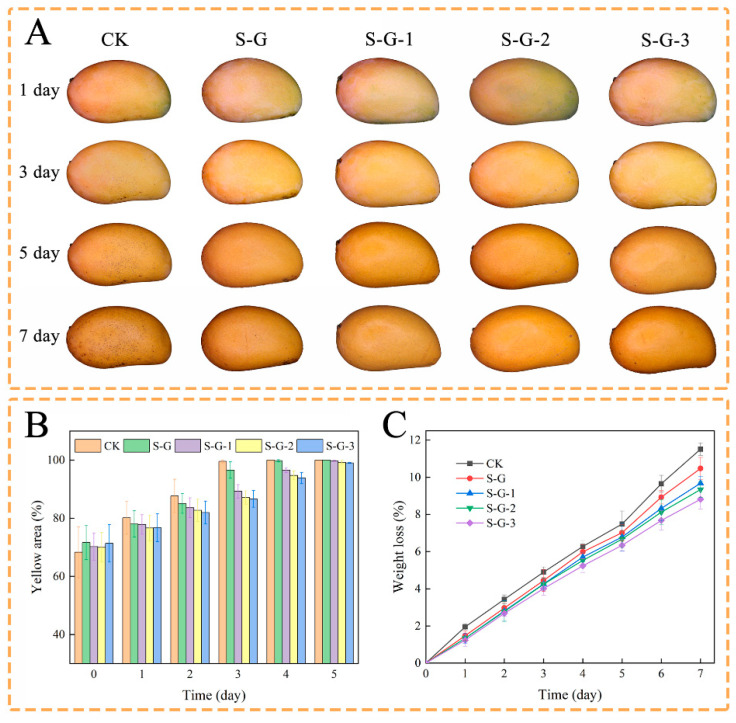
Storage external appearance (**A**), change in yellow area (**B**), and effect of films on weight loss (**C**) of mangoes.

Changes in peel color during storage of the mangoes in each treatment group are shown in Figure 8A,B. The color of mango skin is usually considered as an indicator of mango ripeness. As the mango ripens, the color of the skin changes from green to yellow due to photo-oxidation or enzymatic hydrolysis of chlorophyll [53]. The yellow area of the uncoated mangoes increased faster, black spots appeared on the surface at a later stage, and the increase in the yellow area for the coated mangoes became slower in comparison with the increase in AO concentration, indicating that the film containing AO could slow down the ripening of the mango to a certain extent and prolong the shelf life.

Weight loss is an important feature of the fruit storage process, mainly caused by respiration and water evaporation, which is related to the quality and economic value of the fruit. As shown in Figure 8C, the weight loss rate of all mangoes increased with time. The loss of water in the mangoes resulted in localized softness, which exacerbated enzymatic hydrolysis and accelerated cellular aging, as well as leading to a decrease in nutritional value; this phenomenon was more pronounced during the last three days of observation [54]. This was mainly due to the fact that the AO-containing film acted as a barrier to O_2_, H_2_O, and CO_2_, somewhat reducing the water loss and oxidation of the mango. On the first day, the weight loss of each experimental group was 1.95%, 1.47%, 1.33%, 1.33%, and 1.23%, respectively, and by the seventh day, it was 11.51%, 10.47%, 9.68%, 9.33%, and 8.82%, respectively, which decreased with the increase in the concentration of AO. This indicates that the superior hydrophobicity and antioxidant activity of AO prevented water dissipation from the mango and reduced quality loss [55].

## 4. Conclusions

In this study, SPI was mixed and crosslinked with Gel, to which AO was added to prepare reactive food packaging films for the postharvest preservation of mango by casting method. The results demonstrate that AO has an impact on the films’ mechanical properties, hydrophobicity, and opacity. With increasing AO concentration, different degrees of coalescence of AO droplets could be observed at the microstructural level, which enhanced the films’ roughness and thickness. The incorporation of AO was responsible for the darkening of the films, which also increased the WVP. In addition, the films containing AO had better antioxidant properties, and they had a good preventive effect on *C. gloeosporioides.* Moreover, this shows good freshness retention properties in the practical application of mango storage. This work offers a novel concept for the development of active packaging materials for postharvest preservation of fruit freshness.

## Figures and Tables

**Table 1:** Surface roughness parameters of different films.

Film	R_a_	R_z_	R_q_
S-G	2.50 ± 0.48 ^b^	48.33 ± 2.13 ^b^	3.68 ± 0.66 ^b^
S-G-1	2.69 ± 0.31 ^b^	43.46 ± 8.47 ^b^	3.18 ± 0.58 ^b^
S-G-2	5.41 ± 0.83 ^a^	70.16 ± 9.15 ^a^	7.39 ± 1.56 ^a^
S-G-3	5.71 ± 0.89 ^a^	75.79 ± 8.53 ^a^	7.74 ± 0.78 ^a^

Data are expressed as the mean ± standard deviation (n = 3). Different superscript letters in the same column imply a significant difference (*p* < 0.05).

**Table 2:** Color index values and WVP of films.

Film	L*	a*	b*	ΔE	WVP (×10^−10^ g/m s Pa)
S-G	87.63 ± 0.21 ^a^	−2.13 ± 0.04 ^a^	3.81 ± 0.13 ^c^	6.66 ± 0.16 ^b^	1.09 ± 0.07 ^d^
S-G-1	87.48 ± 0.52 ^a^	−2.21 ± 0.08 ^a^	4.14 ± 0.64 ^b,c^	7.04 ± 0.76 ^b^	1.39 ± 0.08 ^c^
S-G-2	86.80 ± 0.53 ^a,b^	−2.21 ± 0.04 ^a^	4.94 ± 0.66 ^b^	7.99 ± 1.10 ^b^	1.69 ± 0.15 ^b^
S-G-3	85.92 ± 0.26 ^b^	−2.12 ± 0.06 ^a^	6.94 ± 0.22 ^b^	10.17 ± 0.29 ^a^	2.05 ± 0.13 ^a^

Data are expressed as the mean ± standard deviation (n = 5 for color, n = 3 for WVP). Different superscript letters in the same column imply a significant difference (*p* < 0.05).

## Data Availability

The data used to support the findings of this study can be made available by the corresponding author upon request.
